# Role of FGF10/FGFR2b Signaling in Mouse Digestive Tract Development, Repair and Regeneration Following Injury

**DOI:** 10.3389/fcell.2019.00326

**Published:** 2019-12-10

**Authors:** Yu-Qing Lv, Jin Wu, Xiao-Kun Li, Jin-San Zhang, Saverio Bellusci

**Affiliations:** ^1^Key Laboratory of Interventional Pulmonology of Zhejiang Province, Department of Pulmonary and Critical Care Medicine, The First Affiliated Hospital of Wenzhou Medical University, Wenzhou, China; ^2^Institute of Life Sciences, Wenzhou University, Wenzhou, China; ^3^Department of Internal Medicine II, Cardio-Pulmonary Institute, University of Giessen and Marburg Lung Center, Giessen, Germany

**Keywords:** Fgf10, digestive tract organogenesis, stem cells, regeneration, small bowel resection, repair

## Abstract

During embryonic development, the rudimentary digestive tract is initially a tube-like structure. It is composed of epithelial cells surrounded by mesenchymal cells. Reciprocal epithelial–mesenchymal interactions progressively subdivide this primitive tube into distinct functional regions: the tongue, the pharynx, the esophagus, the stomach, the duodenum, the small intestine, the cecum, the large intestine, the colon, and the anus as well as the pancreas and the liver. Fibroblast growth factors (Fgfs) constitute a family of conserved small proteins playing crucial roles during organogenesis, homeostasis, and repair after injury. Among them, fibroblast growth factor 10 (Fgf10) has been reported to orchestrate epithelial–mesenchymal interactions during digestive tract development. In mice, loss of function of *Fgf10* as well as its receptor fibroblast growth factor receptor 2b (*Fgfr2b*) lead to defective taste papillae in the tongue, underdeveloped and defective differentiation of the stomach, duodenal, cecal, and colonic atresias, anorectal malformation, as well as underdeveloped pancreas and liver. Fgf signaling through Fgfr2b receptor is also critical for the repair process after gut injury. In the adult mice, a malabsorption disorder called small bowel syndrome is triggered after massive small bowel resection (SBR). In wild-type mice, SBR leads to a regenerative process called gut adaptation characterized by an increase in the diameter of the remaining small intestine as well as by the presence of deeper crypts and longer villi, altogether leading to increased intestinal surface. Intestinal stem cells are key for this regeneration process. Induction of *Fgf10* expression in the Paneth cells located in the crypt following SBR suggests a critical role for this growth factor in the process of gut adaptation.

## FGF Signaling Ligands and Receptors

The fibroblast growth factor (Fgf) family is composed of 22 members, both in mouse and human (Itoh, 2007). Four Fgf receptors (Fgfr1–4) have been described. The mRNA encoding the first three receptors (Fgfr1–3) undergo alternative splicing thereby generating the so-called IIIb or IIIc isoforms. These isoforms are usually differentially expressed in the epithelium vs. mesenchyme and display different ligand binding capabilities. In addition, a soluble form of FGFR4 was identified in human epithelial cells (Takaishi et al., 2000). During embryonic development and post-natally, multiple Fgf receptors and ligands are detected in the gastrointestinal tract. Fibroblast growth factor receptor 2b (Fgfr2b) ligands (encoded by *Fgf1*, *3*, *7*, *10*) are found in the embryonic as well as the adult mouse intestine [for review see Danopoulos et al. (2017)]. During human development, up to 7 weeks of gestation, fibroblast growth factor 10 (*FGF10*) is detected mostly in the hindgut in the apical side of the epithelium while its expression is found to be decreased at later stages (Yin et al., 2013).

## Digestive Tract Development

During embryonic mouse development, the rudimentary digestive tract is clearly visible at embryonic day (E) 8.0 as a tube-like structure composed of epithelial cells surrounded by mesenchymal cells (Lewis and Tam, 2006). This tube can be divided into foregut (tongue, pharynx, esophagus and stomach, and proximal half of the duodenum), midgut (distal half of the duodenum, jejunum, ileum, cecum, and junction of the proximal two-third and distal one-third of the transverse colon), and hindgut (the distal one-third of transverse colon into the upper part of anal canal). Postnatally, in humans, these demarcations correspond to the areas of arterial supply of the coeliac axis, superior mesenteric, and inferior mesenteric arteries, respectively. Also, the pancreas and the liver develop from the foregut epithelium (Tremblay and Zaret, 2005).

Reciprocal epithelial–mesenchymal interactions progressively subdivide the rudimentary digestive system into distinct functional regions: the tongue, the salivary gland, the esophagus, the stomach, the duodenum, the small intestine, the cecum, the large intestine, the colon, and the anus as well as the pancreas and the liver. The tongue, the salivary gland, and the stomach are normally excluded from reviews dealing with gut development *per se* but as they do represent an important component of the upper part of the digestive system, we also included them in this review. Finally, the liver and the pancreas, through the production of digestive enzymes, are also crucial constituent of the digestive tract.

The normal development of the digestive tract requires interaction between the epithelium and mesenchyme, which involves signaling pathways such as bone morphogenetic proteins (Bmps), Hedgehog (Hh), platelet-derived growth factor alpha (Pdgfa), transforming growth factor beta (Tgfb), Wnts, and Fgfs (Roberts et al., 1995; Karlsson et al., 2000; van den Brink, 2007; McLin et al., 2009). In particular, Fgf10/Fgfr2b signaling plays indispensable roles during digestive tract development where it controls cell proliferation, survival, and differentiation. Due to the focused nature of this review on Fgf10 and its receptor Fgfr2b, we refer the readers to a recent review on the role of multiple Fgf ligands in gut development (Danopoulos et al., 2017).

## Role of FGF10-FGFR2b Signaling in Gut Development and Homeostasis

During E8.0 to E15.5, the proliferation of the mesenchyme as well as epithelium allows the extension of the length of the gut tube and the increase in its circumference to form a stratified epithelium. From E15.5 to E18.5, this pseudostratified cuboidal epithelium differentiates into a simple columnar epithelium. The epithelial layer connects with the underlying mesenchymal layer made of smooth muscle cell and thereby undergoes a morphogenetic process leading to the process of villi formation (Spence et al., 2011). The mechanisms controlling villus morphogenesis at that stage are still unclear.

**FIGURE 1:**
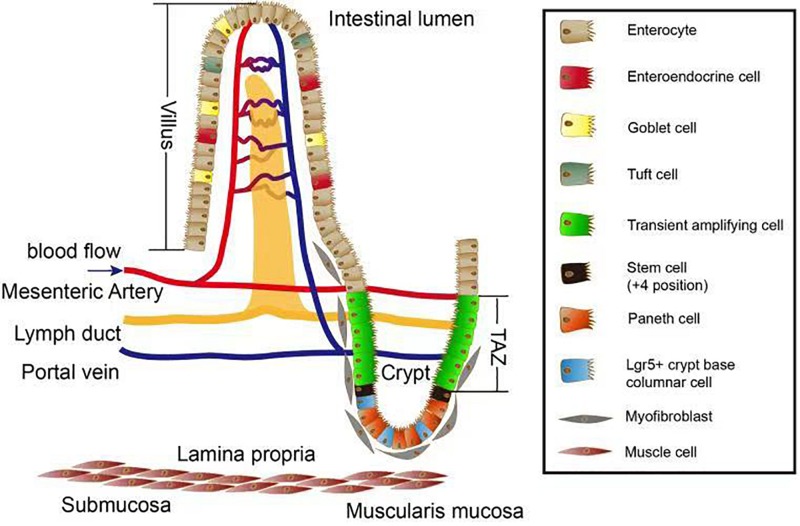
Overview of the intestinal villus/crypt system. Several cell types exist along the crypt–villus axis. Paneth cells, Lgr5+ crypt base columnar cells, and the (+4 position) stem cells together form a crypt stem cell niche which is surrounded by myofibroblasts. After differentiation, the stem cells give rise to different cell types located in villus zone. Stem cells proliferate and become part of the transient amplifying zone (TAZ). These proliferating cells move upward to the top of villus and differentiate into cells important for nutrient absorption such as enterocytes, tuft cells, goblet cells, and enteroendocrine. The lamina propria is where mesenteric artery propria, lymph duct, and portal vein intersect and maintain the homeostasis in terms of blood circulation as well as lymphatic system.

The mesenchymal cells arising from the mesoderm give rise to the longitudinal and circular muscles, as well as the muscularis mucosae and the mesenteric tissue, the function of which is to store fat and allow blood vessels, lymphatics, and nerves to connect with the intestine. The epithelium differentiates to give rise to enterocytes, goblet cells, enteroendocrine cells, Paneth cells, tuft cells, Lgr5+ cells, transient amplifying cells, and crypt base columnar cells (Figure 1).

Fibroblast growth factor 10 plays an important function in gut organogenesis where it modulates proliferation, survival, and differentiation of epithelial cells. The regional expression of Fgf10 indicates a specific regulatory role in these regions of the primitive gut (Fairbanks et al., 2004).

**FIGURE 2:**
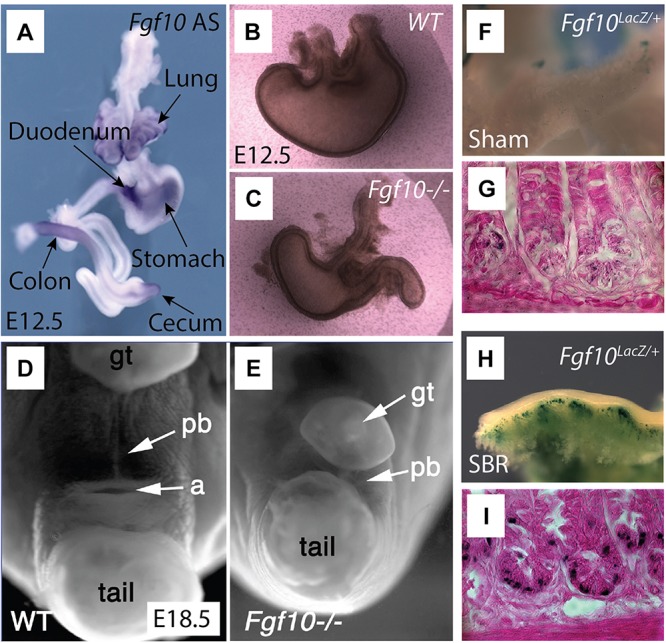
Overview of *Fgf10* expression and associated *Fgf10* KO malformations in the gastrointestinal tract. Adapted under CC-BY 4.0 license, from Burns et al. (2004); Fairbanks et al. (2004), Spencer-Dene et al. (2006), and Tai et al. (2009). **(A)** Whole mount *in situ* hybridization tracing the expression of *Fgf10* (Antisense) in E12.5 gut. *Fgf10* is expressed in the lung, duodenum (arrow points out to the pyloris located at the junction between stomach and duodenum), colon, stomach, as well as cecum. **(B,C)** *Fgf10* KO embryos display stomach dysplasia **(C)** compared to wild type (WT) mouse **(B)**. **(D,E)** *Fgf10* KO embryos **(E)** display smaller perineal body (pb), with indistinguishable anus in E18.5 compared to the WT mouse **(D)**. **(F–I)** A small bowel syndrome (SBR) was induced in *Fgf10*^LacZ/+^ mice. The *Fgf10*^LacZ/+^ mouse displayed increased β-galactosidase (a readout for *Fgf10* expression) in the area of small intestine **(H)** and the crypts **(I)** of the ileum after small bowel resection (SBR) compared to the Sham group **(F,G)**.

In mouse small intestine, *Fgf10* is mostly expressed in the mesenchyme of the duodenum, with low expression in the jejunum and ileum (Figure 2A; Kanard et al., 2005; Nyeng et al., 2011; Al Alam et al., 2015). In human small intestine, FGF10 is detected only in the ileum (Al Alam et al., 2015). *Fgf10* KO mice demonstrated colon, duodenal, and cecal atresia, alongside with anorectal malformations (Figures 2D,E). Furthermore, these animals demonstrated premature cellular differentiation leading to epithelial hypoplasia (Nyeng et al., 2011). In the context of cecum development, there is mesenchymal expansion but no epithelial proliferation in the area where the cecal bud normally forms, between the ileum and large intestine (Burns et al., 2004; Al Alam et al., 2012). It has also been found that Fgf10/Fgfr2b signaling is dispensable for the induction of the rudimentary cecum but absolutely required for epithelial cell proliferation, which is critical for its elongation and development (Burns et al., 2004).

Loss of Fgfr2b function resulting from the trapping of endogenous Fgfr2b ligands by overexpressing a soluble form of Fgfr2b in the adult mouse intestine does not affect cell proliferation or differentiation of the adult gut indicating a dispensable role for Fgfr2b ligands during homeostasis (Al Alam et al., 2015). However, *Fgf10* overexpression in the adult gut leads to increased crypt depths and villus heights throughout the small intestine as well as induction of goblet cell differentiation at the expense of Paneth cells, which decrease in number (Al Alam et al., 2015). Fgfr3 signaling appears also to oppose Fgf10 function in Paneth cells. *Fgfr3* is expressed on the basolateral surface of the epithelium in the lower half of the crypt where the stem cells are located (Vidrich et al., 2004). *Fgfr3* KO mice have decreased number of intestinal crypts compared to controls due to decreased number of stem cells (Vidrich et al., 2009). *Fgfr3* KO mice also display reduced Paneth cell number as well as differentiation, a phenotype also observed upon *Fgf10* overexpression. Altogether, these results suggest that *Fgfr3* signaling regulates the number and differentiation of Paneth cells (Vidrich et al., 2009).

In adult rats, FGF7 treatment enhances cell proliferation and increases goblet cell differentiation (Zeeh et al., 1996; Iwakiri and Podolsky, 2001). The same effect is observed upon *Fgf10* overexpression. This treatment also leads to increased villus height and crypt depth in the intestine (Cai et al., 2013).

Additionally, gain of function experiments suggested that Fgf10 plays a critical role in the proliferation and differentiation of intestinal stem cells and also impacts the supportive cells for these stem cells. The intestinal stem cells are capable of self-renewal as well as giving rise to other functional differentiated cells. Several populations of supportive cells and intestinal stem cells are located at the bottom of the crypt, including Paneth cells (which help sustain and modulate intestinal stem cells by secreting growth factors such as Wnt, Bmps, and Fgf10), crypt base columnar stem cells (CBCs), and +4 stem cells (Potten et al., 2009; Clevers, 2013; Volk and Lacy, 2017).

*Fgf10-*overexpression in mouse−derived organoid units *in vivo* stimulates the formation of tissue−engineered intestine (Torashima et al., 2016). It was also reported that Fgf10 increases goblet cell differentiation by switching the balance with Paneth cells in a model of intestinal enteroids. This process may occur by blocking the Notch signaling which stimulates goblet cell differentiation in concert with apoptosis of Paneth cells. As a result, crypt depth and villus height are significantly increased. In addition, Fgf10 treatment causes an increase in the Mmp7 and Muc2 double-positive transient amplifying cells. This is associated with a concomitant decrease of several stem cell markers, including *Lgr5*, *Lrig1*, *Hopx*, *Ascl2*, and *Sox9* (Al Alam et al., 2015). Paneth cell differentiation and maturation rely on the expression of SRY-box containing gene (Sox9) and Wnt signaling (van Es et al., 2005; Bastide et al., 2007). The precise process of stem cell differentiation in response to Fgf10 signaling needs to be further explored.

## Role of FGF10 in Taste Papillae and Tongue Development

The tongue allows the perception of taste. In mice, tongue morphogenesis starts from E11 with the formation of two tongue buds (Paulson et al., 1985). Subsequently, these buds fuse and form the tongue primordium, followed by proliferation and differentiation in both the epithelial compartment and the mesenchymal compartment (which is made mostly of muscle progenitor cells). At the surface of the epithelial layer, the foliate papillae called taste buds gradually form allowing a mature tongue to develop (Paulson et al., 1985; Burns et al., 2004; Nagata and Yamane, 2004; Nie, 2005).

During mice development, both *Fgfr2b* and *Fgf10* null embryos display thinner and disorganized tongue epithelium as well as impaired taste papillae development (Rice et al., 2004). These results suggest that Fgf10 signaling controls papilla formation (Petersen et al., 2011; Prochazkova et al., 2017).

## Role of FGF10 in Salivary Gland Development

The salivary gland is composed of parotid, submandibular (SMG), and sublingual glands. These allow saliva production in the oral cavity. The saliva contains salivary amylase that displays digestive roles in starch (Mattingly et al., 2015). We refer the reader to published reviews on salivary gland development (Tucker, 2007; Ferreira and Hoffman, 2013). Salivary glands help to produce saliva and maintain oral homeostasis. The salivary gland development depends on reciprocal interactions between the epithelium and mesenchyme. In mice, by E12, the epithelium of the salivary placode in the oral cavity invaginates, at the location where the condensed mesenchyme is found, to form a primary bud. By E13, the process of branching morphogenesis of the salivary glandular buds starts. This allows the formation of a glandular structure at later stages (Patel and Hoffman, 2014). During the branching morphogenesis stage, Fgf10 controls the expression of *Sox9*, which is essential for the establishment of distal progenitor cells as well as for the branching process to occur (Chatzeli et al., 2017). Loss of function of *Fgf10* causes salivary phenotypes with different severities, from failure to form to delayed epithelial branching (Jaskoll et al., 2005; Patel and Hoffman, 2014).

## Role of FGF10 in Stomach Development

Stomach development initiates from the foregut with the formation of a pseudostratified epithelium. At E17.5, the stomach can be subdivided into a forestomach and a glandular stomach. The stratified epithelium in the glandular stomach will further give rise to the corpus and the antrum due to differentiation of the specific progenitors; this includes squamous cells, parietal, and chief and gastric endocrine cells from a pre-patterned gastric progenitor epithelium (Nyeng et al., 2007).

*Fgf10* expression is found at high level of expression ahead of the stomach secondary transition (E15.5–E16.5). *Fgf10* as well as its receptor *Fgfr2b* are expressed in the pre-differentiated mouse stomach at E11.5. Functionally, Fgf10 is part of a network of signaling pathway including Shh and Wnt signaling that synergistically control stomach development. Fgf10 signaling maintains stomach progenitors, morphogenesis, and cellular differentiation and attenuates stomach endocrine terminal differentiation (Spencer-Dene et al., 2006; Nyeng et al., 2007). *Fgf10* KO embryos displayed an underdeveloped, smaller stomach compared to a normal stomach (Figures 2B,C; Spencer-Dene et al., 2006).

Interestingly, overexpression of *Fgf10* affects both gastric epithelial proliferation and differentiation, promotes mucous neck cell differentiation, and suppresses parietal and chief cell differentiation. However, inhibition of Fgfr2b signaling has no impact on epithelial proliferation or differentiation (Speer et al., 2012).

## Role of FGF10 in Pancreas Development

The pancreas, as a digestive glandular organ, originate from the foregut endoderm. Its function is to maintain an appropriate level of blood glucose as well as to contribute to the processing of food digestion inside the gut (Edlund, 1999; Shih et al., 2013). The pancreatic enzymes (trypsin, chymotrypsin, carbohydrates, and lipase) secreted by acinar cells are released for food digestion (Rothman, 1977). At E9.5 in mice, a ventral and a dorsal pancreatic bud emerges from the endoderm. Bud initiation is mediated by sonic *hedgehog* (*Shh*) and *pancreatic and duodenal homeobox 1* (*Pdx1*) in the endoderm (Hebrok et al., 1998). At later stages, the interaction between endoderm and mesoderm leads to the fusion of the two buds. Concomitantly, endocrine cells (acinar cells and ductal cells) as well as exocrine cells (islet cells) start to form (Kumar et al., 2003; Kobberup et al., 2010). High expression of *Pdx1* stimulates endocrine cell differentiation (Lammert et al., 2001), while decreased expression of *Pdx1* or *pancreas transcription factor 1* (*Ptf1a*) causes diabetes, a diseases related to impaired islet cells. These results indicate that *Pdx1* and Ptf1a are crucial for endocrine cells genesis (Kawaguchi et al., 2002; Burlison et al., 2008; Fukuda et al., 2008).

Fgf10 is a mesenchymal-derived growth factor expressed in the pancreas from E9.5 to E12.5. Fgf10 controls the proliferation and differentiation of the adjacent pancreatic epithelial progenitors into functional endocrine and exocrine cell subsets (Bhushan et al., 2001). *Fgf10* allows the formation of *Pdx1*-positive epithelial precursors from the foregut epithelium and its deficiency causes nearly complete loss of endocrine and ductal specification, leading to pancreatic dysplasia (Bhushan et al., 2001; Kobberup et al., 2010). *Fgf10* KO embryos displayed less progenitor cells and fewer exocrine cells differentiation along with malformation of acinar cells (Bhushan et al., 2001). Additionally, in *Fgfr2b* KO embryos, pancreatic ductal cells display less proliferation compared to the corresponding cells in the normal pancreas (Pulkkinen et al., 2003). Mechanistically, this dysplasia phenotype involves in a Sox9/Fgf10/Fgfr2b feed forward loop which is essential to maintain pancreatic organ identity (Seymour et al., 2012).

## Role of FGF10 in Liver Development

The liver is a multifunctional organ that controls saccharolytic and urea metabolism, detoxification, and cholesterol levels as well as digestion. Liver bud genesis starts at E9.0 in mice, with the formation of a diverticulum in the ventral domain of the foregut endoderm. Later on, this diverticulum undergoes de-stratification and proliferation, then invades the surrounding septum, ultimately forming the hepatic buds (Bort et al., 2006). Liver morphogenesis is then initiated by a thickened epithelial structure around the position of the first somite. Hepatic specification requires the interaction of the endoderm with the surrounding cardiac mesoderm septum, transversum, as well as the endothelium (Gualdi et al., 1996; Rossi et al., 2001).

Murine liver bud formation starts at E10 in mice and during the fifth week of gestation in humans. Both hepatoblasts and hematopoietic progenitor cells develop from the liver primordium. Hepatoblasts then differentiate into hepatocytes and cholangiocytes (Migliaccio et al., 1986). Hepatocytes constitute a heterogeneous population displaying different functions in the hepatic lobule. The cluster of periportal hepatocytes allows blood change between the hepatic artery and the portal vein and is linked to the bile ducts. As the fetal hepatic cell mature, glycolytic enzymes secretion is decreased, coupled with increased levels of gluconeogenic enzyme and gain the function of glucogenesis (Devi et al., 1992).

Fgf10 signaling is important to specify the boundaries between the hepatic duct and organs (Dong et al., 2007). *Fgf10* KO mice exhibit smaller livers associated with a decrease in the proliferation and survival of hepatoblasts. As the hepatoblasts undergo proliferation and differentiation to give rise to hepatocytes and cholangiocytes, these results suggest that Fgf10 is crucial in liver genesis and hepatoblast growth via the activation of β-catenin signaling during hepatogenesis (Berg et al., 2007). To date, little is known about the mechanism of action of Fgf10 in hepatic cell proliferation and differentiation during liver development.

## FGFR2b Signaling in Intestinal Injury Repair

*Fgf7* KO mice exhibit increased sensitivity to dextran sulfate sodium (DSS) injury with reduced mucosal barrier repair, suggesting an important role for endogenous Fgf7 in repair after injury (Chen et al., 2002). Recombinant FGF7 treatment has also been tested in the context of DSS-induced colitis injury in rats and mice. FGF7 treatment following colitis induction led to therapeutic benefits such as diminished intestine ulceration and reduced cell death (Egger et al., 1999). Interestingly, in these models, FGF7 delivered before colitis induction was not protective against injury. It is still unclear whether FGF7 can be protective in the context of chemotherapy−induced mucositis (Farrell et al., 2002; Gibson et al., 2002).

By contrast FGF10 (called also Repifermin) elicits both a protective and a therapeutic effect in the model of DSS-induced colitis (Miceli et al., 1999; Greenwood-Van Meerveld et al., 2003). FGF10 also improves the ulceration induced by indomethacin administration in rats, and decreases inflammatory cytokines secretion such as interleukin-6 (IL−6), IL−8, and tumor necrosis factor (TNF) (Miceli et al., 1999; Sandborn et al., 2003; Hamady et al., 2010).

## FGF and Short Bowel Syndrome

Short bowel syndrome (SBS) is synonymous with impaired gut function with decreased absorption of the food associated with severe diarrhea. This syndrome occurs following massive trauma to the gut or following resection of the small intestine due to congenital anomalies. A repair process called adaptation is associated with SBS. This process allows the formation of longer villi and deeper crypts resulting in an increase in the diameter of the remaining small intestine. The process of adaptation results in increased nutrient absorption (Goulet, 1998). In rat models of SBS, recombinant FGF7 treatment led to increased villus height and crypt depth, thickened mucosa, as well as enhanced goblet cell differentiation (Johnson et al., 2000; Washizawa et al., 2004). Interestingly, Fgf10 expression is now detected in the Paneth cells during adaptation while normally absent in the small intestine in mice (Figures 2F–I). These results indicate a crucial role for Fgf10 in intestinal adaptation (Tai et al., 2009). Both gain and loss of function experiments are required to confirm the function of Fgf10 in this regenerative process.

## Ischemia Reperfusion Injury

Following ischemia or hypoxia, the reoxygenation of the gut leads to the so-called ischemia–reperfusion (I/R) injury. During the interruption of the blood supply, the reduced oxygen and nutrients cause oxidative stress and inflammation. This leads to decreased barrier function and potentially to systemic inflammation.

It has been reported that FGF7 treatment elicited a protective effect on intestinal repair in the context of I/R injury. FGF7 treatment rescues cell death and strengthens the barrier function. This is associated with increased recovery of mucosal structures as well as decreased disruption of the tight junctions (Cai et al., 2012). Following I/R injury, FGF7 treatment also increases IL-6 (IL-7) expression, a cytokine which acts on the intraepithelial lymphocytes and which is important for epithelial cell growth (Egger et al., 1998; Cai et al., 2012). Thus, FGF7 treatment is efficient in the repair process of colonic anastomoses via its effects on proliferation, inflammation, and mucus production.

## FGFR2b Ligands and Human Intestinal Diseases

No genetic mutations for the FGF signaling pathway have been identified so far in human with different anorectal malformation or other intestinal defects (Kruger et al., 2008). However, many studies reported a correlation between FGFs expression and gut defects. For example, in patients with inflammatory bowel disease, analysis of gut biopsies indicates that high levels of FGF7 expression are associated with high levels of inflammation (Brauchle et al., 1996; Finch et al., 1996). *FGF7* expression is also high in patients with celiac disease (Salvati et al., 2001) and ulcerative colitis disease (Bajaj-Elliott et al., 1997). In a small subset of colorectal cancer, FGFR2 expression is amplified (Mathur et al., 2014).

## Translational Use of FGF in Intestinal Diseases

Repifermin (also called KGF2 or FGF10) was administered intravenously (IV) to patients with ulcerative colitis. Eighty-eight patients with a diagnostic of ulcerative colitis were enrolled in a clinical trial and received, for five consecutive days, either placebo or escalating doses of FGF10 (1, 5, 10, 25, and 50 μg/kg). Only the lowest dose (1 μg/kg) treatment led to an improvement in the clinical remission at 4 weeks after treatment (Sandborn et al., 2003). The readouts focused on the endoscopic appearance, stool blood component, and stool frequency. While all the FGF10 doses used were well tolerated, the higher doses did not trigger a positive outcome. This observation suggests that higher doses of FGF10 could trigger an inhibitory effect on cell survival and proliferation. The fact that the lower dose used led to a beneficial effect suggests that follow-up clinical trials should be done with lower doses of FGF10.

## Where Do We Go From Here?

One of the biggest problem for the translational use of FGFs is that while they work very well in pre-clinical models, they fail to elicit a positive effect in the few and limited clinical trials. In particular, given the importance of Fgf10 during digestive tract development, the use of recombinant FGF10 in humans has been disappointing in the limited results published to date. Key aspects to consider for future studies are the stability, dosage, and the route of administration of this growth factor to ensure effective repair. Considering the important role played by FGF signaling in development and repair, it will be worth the effort to optimize the experimental conditions for the efficient use of FGFs to treat patients with digestive tract diseases.

## Author Contributions

SB, J-SZ, and X-KL conceived the study. Y-QL and JW wrote the manuscript. J-SZ and SB critically revised the manuscript and acquired funding.

## Conflict of Interest

The authors declare that the research was conducted in the absence of any commercial or financial relationships that could be construed as a potential conflict of interest.
